# Commentary: Association between wine consumption and cancer: a systematic review and meta-analysis

**DOI:** 10.3389/fnut.2024.1335731

**Published:** 2024-02-21

**Authors:** Fausta Natella, Gianni Pastore, Raffaella Canali

**Affiliations:** Consiglio per la Ricerca in Agricoltura e l'Analisi dell'Economia Agraria (CREA), Food and Nutrition Research Centre, Rome, Italy

**Keywords:** alcohol, wine, cancer, meta-analysis, spin

Reading the article by Luceron-Lucas-Torres et al. (1) we were quite surprised to find that in the abstract the authors drew conclusions that are not supported by data reported in the paper.

In the abstract (but also in the text), in fact, authors stated that “wine drinking demonstrated a protective trend regarding the risk of developing pancreatic, skin, lung, and brain cancer as well as cancer in general”. This statement is puzzling, considering that authors themselves summarize their results concluding that the “systematic review and meta-analysis revealed no association between wine consumption and general, upper digestive tract, colorectal, renal, pancreatic, skin, lung, brain, and gynecological cancers” and that “The study findings reveal no association between wine consumption and the risk of developing any type of cancer”.

Moreover, specifically referring to pancreatic, skin, lung, brain, upper digestive tract and general cancer, in the meta-analysis results section it is clearly reported that “the number of included studies focusing on these cancers was insufficient to perform a meta-analysis”. In our knowledge, according to the scientific approach, if there is no data sufficient to correctly perform a statistical analysis, then those data are not sufficient to draw any scientifically based conclusion.

Furthermore, the meta-analysis quality is poor, it is in fact burdened by the presence of both substantial heterogeneity and publication bias, that were neither investigated nor included as limitation of the study; moreover, and again more important, it is not clear, because never reported, which groups were compared (drinkers vs. non-drinkers?) and why the exposure level (dose of wine consumption) was not taken into any account.

The article contains improper citations, but above all, it is distorted by the diffuse use of “spin” (the misrepresentation of a study's actual results) (2) and contains a lot of misleading messages about the possible positive effect of wine. Just as a representative example: authors stated that “many components in wine could have anticarcinogenic effects, such as resveratrol, which plays antioxidant, antimutagenic, and antiinflammatory roles in carcinogenesis […] Other components with anticarcinogenic properties anthocyanins, quercetin, and tannins”. Also considering that the anticarcinogenic effects of these compounds have only been observed in *in vitro* studies, we cannot understand the rationale behind the authors mention the presence of negligible quantities of compounds with presumed anti-carcinogenic action, forgetting to mention the presence of over 10% by volume of ethanol, classified by The International Agency for Research on Cancer (IARC) as a Group 1 carcinogen, that is certainly carcinogenic to humans.

We think that any misleading message about unproven protective effect of wine consumption on cancer is extremely dangerous and could contribute to spread scientifically incorrect information and wrongly induce people to drink wine to protect themselves against cancer.

Consolidated scientific literature demonstrates that the consumption of alcoholic beverages (any type) increases the risk of cancer (mouth, pharynx, larynx, esophagus, liver, colorectum and breast) (3). Alcohol accounts for an estimated 741,300 (or 4%) of new cases of cancer worldwide and contributes to nearly 400,000 deaths due to cancer every year (4). All cancer prevention guidelines agree in communicating that there are no levels or quantities of consumption of any type of alcoholic beverage considered “safe” for health, providing as a general indication the limitation of the consumption of any alcoholic beverage and specifying that for preventing cancer the best choice is not to drink^1^^−^^3^.

Due to the consistent use of spin and misrepresentation of study results to positively influence wine consumption, retraction of the paper should be considered.

## Author contributions

FN: Conceptualization, Writing – original draft, Writing – review & editing. GP: Writing – review & editing, Conceptualization. RC: Writing – review & editing, Conceptualization.

## Footnotes

^1^WCRF/AICR. Available online at: https://www.wcrf.org/diet-activity-and-cancer/cancer-prevention-recommendations/limit-alcohol-consumption/.

^3^European code against cancer International Agency for Research on Cancer (IARC)/World Health Organization (WHO). Available online at: https://cancer-code-europe.iarc.fr/index.php/en/.
